# Use of the Maslach Burnout Inventory Among Public Health Care Professionals: Scoping Review

**DOI:** 10.2196/44195

**Published:** 2023-07-21

**Authors:** Juliana Pontes Soares, Rayssa Horácio Lopes, Paula Beatriz de Souza Mendonça, Cícera Renata Diniz Vieira Silva, Cláudia Cristiane Filgueira Martins Rodrigues, Janete Lima de Castro

**Affiliations:** 1 Postgraduate Program in Collective Health Federal University of Rio Grande do Norte Natal Brazil; 2 Postgraduate Program in Collective Health Federal University of Espírito Santo Vitória Brazil; 3 Technical School of Health of Cajazeiras Federal University of Campina Grande Cajazeiras Brazil; 4 School of Health Federal University of Rio Grande do Norte Natal Brazil

**Keywords:** burnout professional, burnout, health care professional, health personnel, health professionals, Maslach Burnout Inventory, mental health, occupational health, public health services, public health, workplace stress

## Abstract

**Background:**

Work can be considered a source of living, well-being, and socioeconomic development. When the work environment negatively influences individuals, it may trigger emotional disturbances, behavioral problems, chronic stress conditions, and illnesses such as burnout syndrome (BS). Recently, studies on BS have increased and placed a special focus on health care professionals. The prevalence of BS among health professionals is associated with their chronic exposure to human hardship and long working hours without proper rest. These factors have contributed to greater stress and high physical and emotional exhaustion levels.

**Objective:**

This study aims to identify and map studies using the Maslach Burnout Inventory (MBI) scale to identify burnout syndrome in health professionals working in public health services.

**Methods:**

This scoping review was developed based on the Joanna Briggs Institute (JBI) Reviewers Manual and reported according to the PRISMA-ScR (Preferred Reporting Items for Systematic Reviews and Meta-Analyses Extension for Scoping Reviews). A total of 6 databases were searched to identify relevant studies: Embase, LILACS, MEDLINE or PubMed, PsycInfo, Scopus, and Web of Science. Gray literature was consulted on ProQuest Dissertations and Theses Global, Google Scholar, Brazilian Digital Library of Theses and Dissertations, and Open Access Theses and Dissertations. Additionally, the reference lists were searched to retrieve studies not previously selected. The steps followed in this study were proposed by Arskey and O’Malley and Levac, Colquhoun, and O’Brien: identification of research questions, identification of potential studies, study selection, data extraction and imputation, data analyses and interpretation, and consultation with stakeholders. The detailed methodology was published in a protocol.

**Results:**

A total of 55 articles were identified after screening for eligibility criteria, published between 1999 and 2021 in 32 countries. Most reports were published in Brazil, Spain, and China. A total of 22 versions of the MBI were identified, presenting different items, scores, and cutoff points. The included studies had recommendations and implications for clinical practice. The consultation with stakeholders allowed knowledge translation for those interested in BS.

**Conclusions:**

Studies mostly included physicians (34/55, 61.8%) and nurses (24/55, 43.6%), and the original version of MBI was predominantly used. Divergences in BS classification were highlighted, which may be related to MBI cross-cultural adaptations and applications in other countries. This study contributes to the advancement of research regarding burnout syndrome as an occupational illness since it has harmful consequences for workers, health care services, and the quality of care provided to the population.

## Introduction

Work can be considered a source of living, well-being, and socioeconomic development. When the work environment negatively influences individuals, it may trigger emotional disturbances, behavioral problems, chronic stress conditions, and illnesses such as burnout syndrome (BS) [1-3]. This term was coined by the psychoanalyst Freudenberger [4] after studying unwanted emotional and interpersonal exhaustion factors that affected people working in health care services [4]. Later, the social psychologist Maslach studied emotions in the workplace and their relationships with illness. The initial study indicated that clinical symptoms of BS were associated with the mental health and social status of the care provider and recipient in the occupational context [1].

The World Health Organization incorporated BS in the latest version of the International Classification of Diseases (ICD-11) as an “occupational phenomenon” resulting from poor management of a demanding work environment [5,6]. Recently, studies on BS have increased and placed special focus on health care professionals [3,7-9]. The prevalence of BS among health care professionals is associated with their chronic exposure to human hardship and long working hours without proper rest. These factors have contributed to greater stress and high physical and emotional exhaustion levels [3,6,10,11].

The public health care workforce is mentioned as the most exposed to BS [11]. Prolonged psychophysical distress, emotional exhaustion, and a lack of personal accomplishment negatively influence occupational performance and mental health. In addition, occupational illness is globally recognized as a public health problem because it encompasses dimensions that go beyond health. High rates of absenteeism, job turnover, and social security expenses also impact the socioeconomic dimension [10,11].

The assessment of BS encompasses a systematic approach based on psychometric variables of a scale conceived by Maslach and Jackson [12], called the Maslach Burnout Inventory (MBI). The MBI is a self-administered scale composed of affirmative sentences about feelings and attitudes toward work. It has 3 dimensions: emotional exhaustion, depersonalization, and a lack of personal accomplishment. This multidimensional model adopted by Maslach and Jackson is the most cited in the literature about BS [1-3,13].

The version published in 1981 was progressively updated, translated, and adapted to meet the needs of different professional groups worldwide [1-3]. The study conducted by De Hert [3] presented 5 versions of the MBI scale: Human Services Survey (MBI-HSS), Human Services Survey for Medical Personnel (MBI-HSS-[MP]), Educators Survey (MBI-ES), General Survey (MBI-GS), and General Survey for Students (MBI-GS [S]). The high number of adapted scales is due to the diverse occupational sectors and the attempt to find meaningful solutions to prevent occupational illness [3,7,8]. Studies using the MBI provided evidence to guide managers and other health authorities in planning, implementing, and evaluating interventions [3,5,7,9]. Moreover, these studies helped develop guidelines to stimulate ideas on workplace organization and improve the physical and mental well-being of health professionals [3,5,7,9].

Currently, a gap concerning mapping the use of MBI among health professionals working in public services has been identified. The MBI is mentioned in the literature as the gold standard for evaluating BS [10], and despite its importance, its use is not standardized. This makes it difficult to compare studies, even when they are developed from the same perspective. Thus, the results of this scoping review may direct initiatives to think about a possible standardization in the use of the MBI, contributing to the advancement of research aimed at understanding the relationship between work and mental illness among professionals. Considering the importance of MBI and the exposure to occupational factors that may lead to mental suffering, this scoping review aimed to identify and map studies using the MBI scale to identify BS in health professionals working in public health services.

## Methods

### Study Methodology

**Figure 1:**
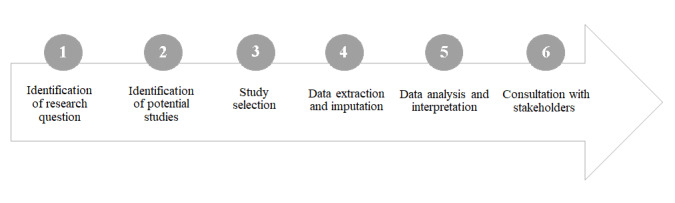
Steps of the scoping review.

This scoping review was developed based on the Joanna Briggs Institute Reviewers Manual (JBI) [14] and reported according to the PRISMA-ScR (Preferred Reporting Items for Systematic Reviews and Meta-Analyses Extension for Scoping Reviews) [15]. The steps followed in this study were proposed by Arskey and O’Malley [16] and Levac et al [17] (Figure 1). The detailed methodology was published in a protocol [18]. The main changes in this review were related to the analysis of the latent profile, the form of application of the scales, and the challenges and limitations that were not identified in the included studies.

The research question was developed based on the acronym PCC (Population, Concept, Context) [14]: P–Public health care professionals; C–Maslach Burnout Inventory; and C–BS in health care professionals. The following research questions were identified: (1) How is MBI used to identify BS in health care professionals working in public health services? (2) What is the most studied professional category using MBI? (3) What are the main results of MBI in health care professionals working for public health services? And (4), what are the recommendations for clinical practice arising from the use of MBI?

A total of 6 databases were searched to identify relevant studies: EMBASE, LILACS, MEDLINE or PubMed, PsycInfo, Scopus, and the Web of Science. Gray literature was consulted on ProQuest Dissertations and Theses Global, Google Scholar, Brazilian Digital Library of Theses and Dissertations, and Open Access Theses and Dissertations. Additionally, reference lists were searched to retrieve studies not previously selected.

Inclusion criteria encompassed quantitative and qualitative studies that used the MBI scale to identify BS in health care professionals working in public health services, full-text availability, and no language or date restriction. Exclusion criteria encompassed duplicate publications, literature reviews, letters, editorials, theoretical essays, and opinion articles; studies that analyzed BS in other professionals, professionals working in private services, or undergraduate students; studies that used different scales, did not isolate the MBI in the results section, or did not use the MBI to investigate BS; and studies with an unavailable full-text version.

Searches and screenings occurred from February 2 to April 6, 2022. The search strategy was developed using 4 controlled vocabularies (DeCS, MeSH, EMTREE, and APA THESAURUS) [18]. The extraction, conversion, combination, construction, and use model [19] was used and refined with the help of a librarian. Natural and controlled languages were chosen for greater sensitivity and expansion of search results [19].

Selected studies were exported to a Microsoft Excel spreadsheet, and duplicates were manually excluded. A total of 2 independent researchers screened the titles and abstracts, and a third reviewer was consulted to resolve possible disagreements. As recommended by the JBI Manual [14], a pilot study was conducted to evaluate the consistency of the protocol [18] and select studies according to titles and abstracts. Subsequently, full texts were retrieved, and reference lists of the included studies were analyzed.

Data extraction and imputation ensured the consistency and reliability of the results. A total of 2 independent researchers used a data extraction sheet, adapted based on the JBI model [14], containing the following: study, type of study, year of publication, the context of publication, journal, study aims, study design, population, sample, MBI version, MBI domains (emotional exhaustion, depersonalization, and lack of personal accomplishment), number of items, type of Likert scale, cutoff point, results, challenges and limitations in using the scale, and implications for practice.

Quantitative variables (type of publication, year, country, study design, studied professional category, scale versions, domains, number of items, and Likert and cut-off points) were analyzed descriptively and presented as absolute and relative frequencies. Qualitative variables related to recommendations and implications for practice were processed using the IRAMUTEQ software [20]. Textual fragments extracted from the results and conclusion sections produced a text corpus that was further analyzed using similarity analysis.

The results of this review were presented to 7 stakeholders (researchers with experience in the use of MBI and BS), who answered questions regarding the findings in compliance with Levac et al [17]. This procedure intends to exchange knowledge and develop strategies for disseminating results and ideas for future studies. The following questions were asked:

1. In the scientific field, disseminating research findings is very important for academia, health services, management, health professionals, and the entire community. Therefore, we would like your suggestions on ways to disseminate the results of this study.
2. How do you think this study could contribute to standardizing the use of MBI?
3. Based on this scoping review, what ideas would you have for future studies?

### Ethics Approval

Given the participation of stakeholders, the study was submitted and approved by the research ethics committee of the Onofre Lopes University Hospital of the Federal University of Rio Grande do Norte (No. 4,952,319 and CAAE No. 46284921.4.0000.5292) on September 3, 2021.

## Results

**Figure 2:**
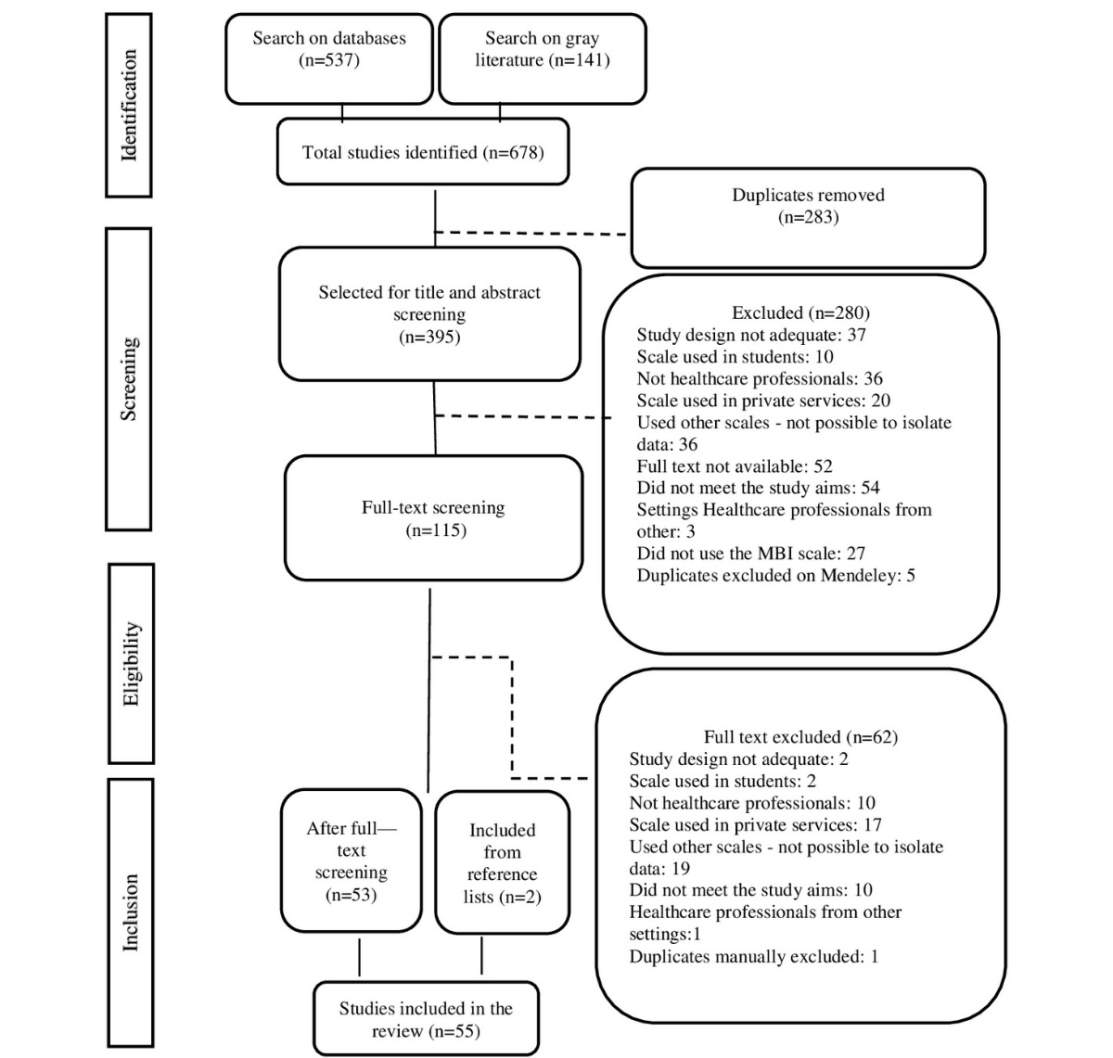
Flowchart of study selection adapted from the PRISMA (Preferred Reporting Items for Systematic Review and Meta-Analyses).

A total of 678 publications were identified, of which 537 were peer-reviewed studies and 141 were retrieved from gray literature. After screening and eligibility criteria, 53 studies were eligible, and 2 studies were identified after consulting the reference lists; thus, 55 studies were included in this scoping review (Figure 2).

### Characteristics of Included Studies

A total of 55 scientific articles published between 1999 and 2021 were included; most were published between 2016 and 2021 (n=32, 58.1%), mainly in 2018 (n=8, 14.5%) (Multimedia Appendix 1).

**Figure 3:**
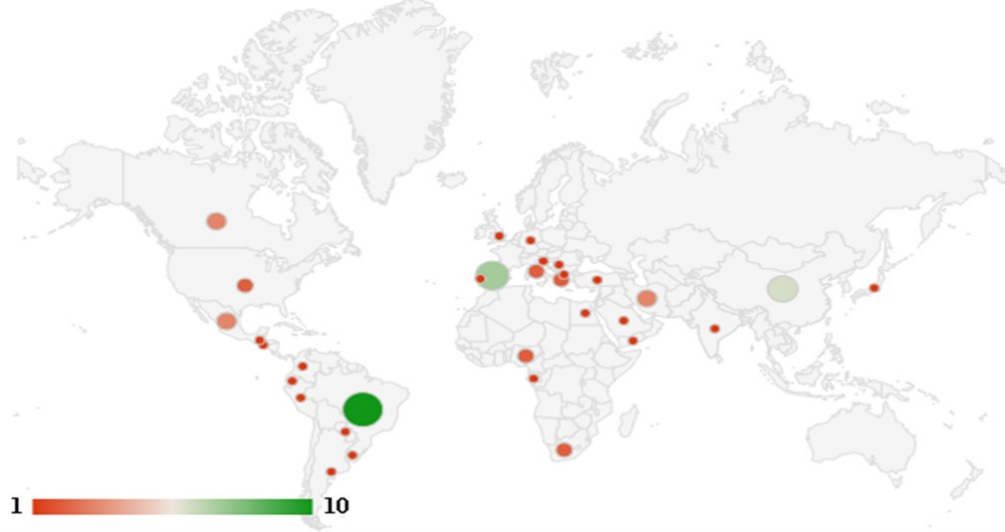
Distribution of studies according to country of origin.

Studies were developed in several countries, including Brazil (n=10, 15.8%), Spain (n=7, 11.1%), and China (n=6, 9.5%) (Figure 3). For mapping purposes, we adopted the total number of countries (n=63) since 1 study was developed in more than one country.

The study design was reported in 44 (80%) studies; most were cross-sectional (n=37, 67.2%). Studies assessed a single health profession (n=40, 72.7%) or more than one category (n=15, 27.2%). The MBI scale was predominantly used to assess physicians (n=34, 61.8%) and nurses (n=24, 43.6%).

### Characteristics of the Maslach Burnout Inventory

A total of 21 versions of the MBI were identified. The original version was the most used (20/55, 36.3%) [20-39] as shown in Table 1.

The MBI scale comprises 3 domains: emotional exhaustion, depersonalization, and lack of personal accomplishment. Most studies (53/55, 96.3%) analyzed all 3 domains [21-73].

A total of 42 (76.3%) studies used an MBI scale composed of 22 items [21-32,34-39,41,42,45,46,48-58,60,62-64,66,68,69,​^71^,^72^]. Additionally, 11 (20%) studies did not describe which MBI items were used [33,43,47,59,61,65,68,70,73-75].

Answers to the MBI were distributed on a Likert-type scale, and most studies used a 0 to 6 score (26/55, 47.2%) [21,23-25,28,30-32,36,37,41-44,48,49,51,54,57-59,62,64,66,67,69,74]. A total of 16 (29%) studies did not describe how they rated the scores [22,27,33,38,46,47,50,53,55,56,61,65,68,71,75].

Studies used different ways to classify the scores of each MBI domain. The most used cutoff point (10/55 studies, 18.1%) established high (≥27 points), medium (from 19 to 26 points), and low scores (≤19 points) for emotional exhaustion. Depersonalization was considered high (≥10), medium (from 6 to 9), and low (≤6). Personal accomplishment was also represented as high (≤33), medium (from 34 to 39), and low (≥40) [27,31,34,38,58,59,61,65,71,75].

**Table 1:** Distribution of Maslach Burnout Inventory (MBI) versions used in the included studies.

Version of the MBI	Studies, n (%)
MBI original	20 (36.3)
MBI-HSS^a^	9 (16.3)
Spanish version of MBI	3 (5.4)
Spanish version of MBI-HSS	3 (5.4)
Chinese version of MBI-GS^b^	2 (3.6)
Chinese version of MBI-HSS	2 (3.6)
Short version of MBI	2 (3.6)
French version	1 (1.8)
Persian version	1 (1.8)
Brazilian version	1 (1.8)
Greek version	1 (1.8)
Portuguese version	1 (1.8)
Japanese version	1 (1.8)
English version translated to Arabic	1 (1.8)
German version	1 (1.8)
South African version	1 (1.8)
Turkish version	1 (1.8)
French version for physicians	1 (1.8)
Version validated by Robayo Tamayo	1 (1.8)
Brazilian version validated by Lautert	1 (1.8)
Version validated and adapted by Benevides-Pereira	1 (1.8)

^a^MBI-HSS: MBI Human Services Survey.

^b^MBI-GS: MBI General Survey.

### Recommendations and Implications for Practice: Similarity Analysis

A text corpus was developed based on information from the included studies to analyze the recommendations and implications for practice. The text corpus underwent a lexicographic analysis using the IRAMUTEQ software 19 to generate the similarity analysis. The analyzed text corpus consisted of 49 texts (87 text segments) and a 64% usage rate (2933 occurrences and 875 forms). In the similarity analysis, the cutoff point was adopted as the triple of the quotient between occurrences and forms; thus, words with a frequency of ≥10 showing theoretical coherence with the research topic were inserted.

**Figure 4:**
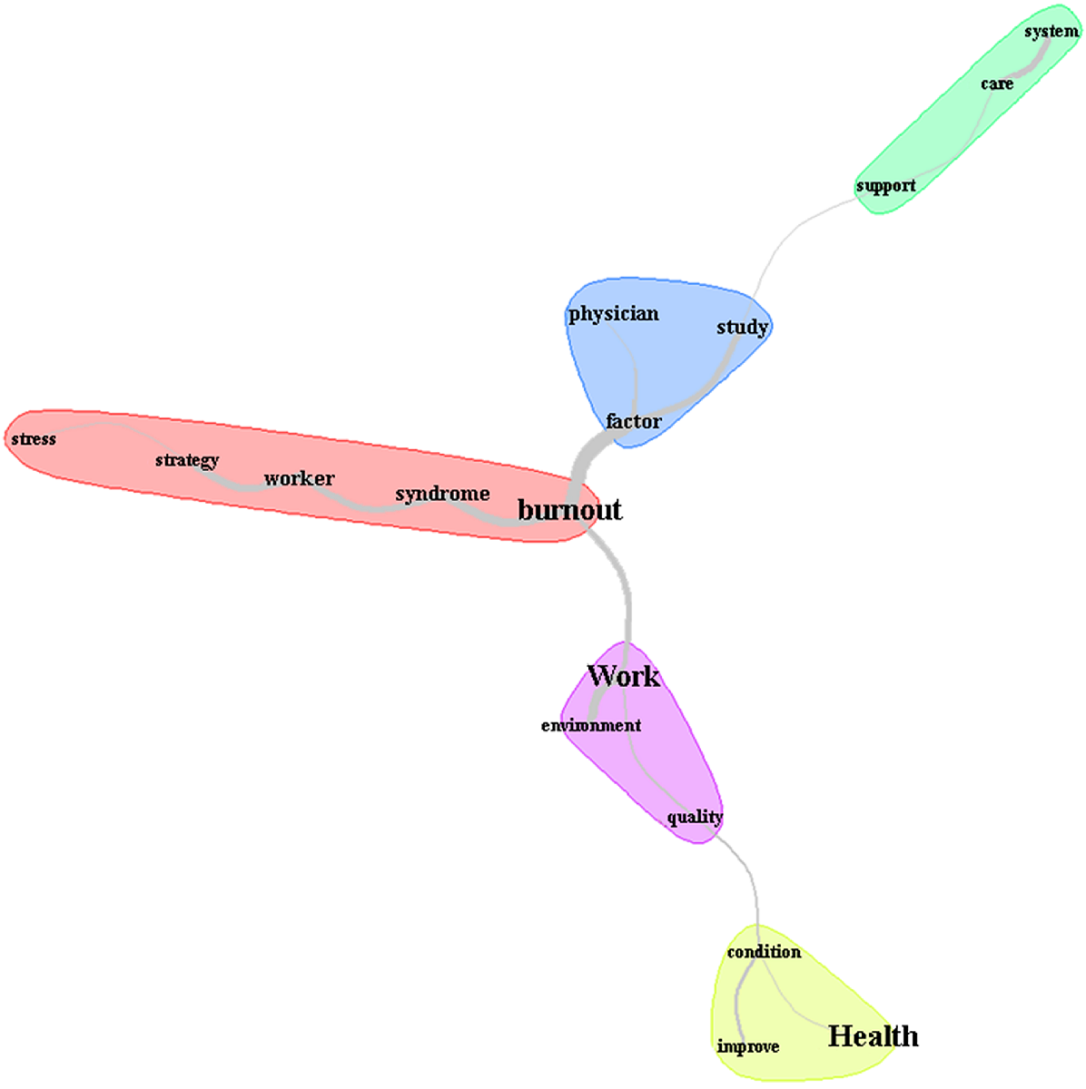
Image created from the similarity analysis with recommendations and implications for practice based on using the Maslach Burnout Inventory (MBI) scale. Fruchterman Reingold presentation, chi-square score with community and halo.

The image created was composed of 5 community halos (Figure 4) that highlighted the words “health,” “work,” “burnout,” and “physician.” The central community was formed with the word “burnout,” branching into “syndrome,” “worker,” “strategy,” and “stress.” The central community branches into 2 communities in the upper portion. The first includes the words “physician,” “factor,” and “study,” and joins with the words “system,” “support,” and “care.” In the lower portion, the central community also branches into 2 communities, revealing the words “work,” “environment,” and “quality,” joining with “health,” “condition,” and “improve.”

According to the included studies, recommendations and implications for practice addressing BS require strategies focused on stress management and strengthening the health systems. Health systems should develop studies regarding factors in the work environment impacting the quality of care. This approach could improve the health conditions of professionals, especially physicians.

The included studies did not report limitations or challenges in using the MBI. Also, a causal relationship between events was not established because most study designs were cross-sectional.

## Discussion

### Main Findings

Mapping the literature on the Maslach Burnout Inventory allowed for the observation of ways to identify the presence of BS in professionals working in public health services. Several studies using the MBI were developed in Brazil, a country of continental dimensions and cultural diversity that challenges the health status of professionals [76]. The structural challenges in the public Brazilian Unified Health System (SUS) have been marginally addressed, often in a fragmented and superficial way [77].

Most professionals within the SUS face conditions including precarious and fragile employment contracts, low wages, a high workload due to a lack of complete teams, poor working conditions and infrastructure, and a lack of supplies and equipment. The sum of these conditions compromises the health status and may result in physical and mental illness [77-79]. Work-related mental disorders, including BS, may be associated with work organization in the health field. This relationship could explain the high number of studies conducted in Brazil and identified in this review [29,31,35,38,52,55,63,71-73].

Spain was the second country in the number of studies included in this review [21,34,45,46,48-50]. Research shows that health systems in Brazil and Spain have similarities linked to the implementation of national health systems, organizational principles, universal coverage, and reorganization involving primary health care. Also, both countries face operational challenges in ensuring universality, dealing with budget cuts, and managing the expansion of private health insurance [80,81].

Studies using the MBI for assessing BS focused most on physicians. Literature reports a prevalence of BS close to or higher than 50% among these professionals, especially for those involved with direct patient care [82-86]. BS negatively impacts patients, health organizations, and systems, increasing the risks of errors during medical procedures [82,84,87]. The predictors of BS among physicians include high workload (eg, long and stressful night shifts), work conflicts, increased digitization and work bureaucracies, and a lack of continued education, support from colleagues, and autonomy at work [82-84,88]. The second professional category most present in the studies was nursing. These professionals are also exposed to a greater workload as well as emotional stressors because they are closer to patients and because they often work in different work environments despite the low wages offered to the category. This sum of factors results in negative impacts on the mental health of this workforce, contributing to the development of BS [89].

Among the selected studies, physicians and nurses worked mostly in primary health care (PHC) services [22,29,35,36,42,63,65]. It is known that this level of care is considered the gateway to health systems and therefore receives a greater demand from users who seek the service because it is closer to their homes. This greater demand overloads professionals, leaving them susceptible to the development of BS [62].

Regarding the type of study, a cross-sectional design was predominantly used. This design favors the description of specific population characteristics at a single point in time, contributing to a representative sample of the population. Advantages of the cross-sectional design include low cost, low risk of data loss, and identification of the prevalence of a certain phenomenon. However, this design does not establish a causal relationship between events [90,91].

Although the MBI neither identifies antecedent nor consequent factors linked to BS, it allows the identification of suggestive or onset signs of the syndrome [92]. Despite its general use, no consensus on how to interpret the MBI is currently available, justifying the differences in the scoring and description of results among studies. Variations in the frequency and cutoff points of the scale require accurate description in each study to avoid divergences in the results [6,93]. Most studies included in this review used the score (Likert-type scale and number of items) corresponding to the original version of the MBI. However, several studies did not follow the cutoff points of the MBI guidelines, which include high scores in the subscales of emotional exhaustion and depersonalization and low scores in personal performance. Studies mostly considered high scores in all 3 subscales as indicative of BS [27,31,34,38,​^58^,^59^,^61^,^65^,^71^,^75^].

In addition to adapted, translated, and validated versions, an Indian study translated the MBI into 3 indigenous languages to better fit the studied population [74]. The various ways of interpreting the MBI show that some authors consider the tridimensionality (ie, high scores for emotional exhaustion and depersonalization and low scores for personal performance) for the BS outcome [12]. Other authors consider only 1 dimension, regardless of which shows the higher scores [94], or 2 dimensions (high scores for emotional exhaustion and depersonalization) [95]. In this review, most studies used tridimensionality as the BS outcome, as recommended by the original version.

The cross-cultural adaptation of the MBI is crucial, but differences in interpreting the scale hamper the comparison among studies [6,93]. One study used previously described cutoff points in a population, thus, allowing the comparison of results with other publications [63]. Another study used the cutoff points recommended by the MBI guideline to enable international comparison [21].

BS challenges health care systems worldwide, affecting between 25% and 75% of health care professionals [96]. The prevalence also varies between countries, professional specialties, and work sectors [96]. Regarding recommendations and implications for practice arising from the use of MBI, our results highlight the importance of a holistic view of health for professionals working in public health services. Furthermore, the findings support the implementation of prevention programs that should be based on a set of individual and organizational strategies.

Implementing strategies that minimize occupational stress may contribute to reducing BS in health services. Strategies must be aimed at the worker individually and collectively in an organizational way. The reorganization of the work process, staff sizing according to the needs of the demands, distribution of activities according to the response capacity, recognition of the first signs that characterize BS, modification of stressors, elaboration and implementation of organizational policies that improve the quality of life and offer emotional support, as well as the inclusion of integrative and complementary practices [97-99], are strategies that may positively impact the health and well-being of health professionals and improve the quality of care for patients seeking public health services worldwide.

### Stakeholder Consultation

When asked about ways of disseminating our research findings, stakeholders reported the dissemination of the results could be performed in the following ways: scientific articles, including graphs and tables; in health centers, support centers for health workers, and with health care managers; social media networks (ie, connecting researchers in teaching and management institutions); indexed journals in international, open, and peer-reviewed databases, printed or web-based; and scientific events.

In response to the contributions of this review to standardizing the use of MBI, participants answered that the scale was broad and reliable, and its standardization would be important for allowing comparisons among studies. They also reported that standardizing the use of MBI was needed to critically evaluate and compare the different versions and indicate their advantages and disadvantages.

Regarding suggestions for future studies, participants emphasized the importance of developing studies with primary health care professionals and professors since they are exposed to several psychological risks but are little studied. Participants highlighted that the scale was used exclusively to assess BS and did not consider the antecedents or consequences of the syndrome. Therefore, studies on different ways of measuring the predisposition to BS are needed. Future studies should also include a systematic review with meta-analysis and provide robust data on the profiles of participants included in studies using the MBI.

### Strengths and Limitations of the Study

This scoping review identified and mapped the use of the MBI scale among professionals in public health services. We expect an increase in the number of publications on the use of MBI in professionals working in these settings, especially cross-sectional studies, as they are important to explore local contexts.

This research followed the methodological recommendations proposed by the JBI and was conducted without date or language limits to allow a broad inclusion of the literature. Furthermore, we shared the main results with 7 specialists in the area, favoring knowledge transfer with interested parties [17].

A limitation of this study is that some relevant databases may have been missed despite extensive searches. Moreover, although we double-checked the data analysis, loss of information may have occurred during the translation of the studies into Portuguese (ie, the native language of the authors). Another possible limitation concerns the inclusion of public health professionals in the selection of studies. We highlight the difficulty of identifying this workforce in studies where it was not clearly stated that it was a public health service, and therefore some important information may not have been included due to the exclusion of studies that did not show the scenario in which the study was conducted.

### Conclusions

We identified and mapped the use of the MBI scale among professionals from public health services in studies from different countries, mainly performed with physicians and nurses. Among different versions of the Maslach Burnout Inventory, the original instrument is the most widely applied to study BS in health professionals from public health systems.

The findings identified divergences regarding how studies assessed BS, probably due to cross-cultural adaptations of the scale in different countries. The study contributes to the advancement of research on BS as an occupational illness since, when answering the MBI, the participants refer to situations experienced in their work environment, reinforcing the relationship between the stressors present in the work environment and the development of BS, which characterizes it as an occupational disease that can have consequences for workers, for health services, and for the quality of the assistance provided to the population.

## Appendix

Multimedia Appendix 1 Description of included studies.

## Abbreviations

BS: burnout syndrome
ICD: International Classification of Diseases
JBI: Joanna Briggs Institute
MBI: Maslach Burnout Inventory
MBI-ES: MBI-Educators Survey
MBI-GS: MBI-General Survey
MBI-GS (S): MBI-General Survey for Students
MBI-HSS: MBI-Human Services Survey
MBI-HSS-(MP): MBI-Human Services Survey for Medical Personnel
PCC: Population, Concept, Context
PHC: primary health care
PRISMA-ScR: Preferred Reporting Items for Systematic Reviews and Meta-Analyses Extension for Scoping Reviews
SUS: Unified Health System
