# Supplementary material for: Use of the Maslach Burnout Inventory Among Public Health Care Professionals: Scoping Review
Source: JMIR Ment Health. 2023 Jul 21;10:e44195. doi: 10.2196/44195 (PMC10403803; doi:10.2196/44195)
Supplement: Multimedia Appendix 1 [file mental_v10i1e44195_app1.docx]

**Multimedia Appendix 1**. Description of included studies.

| **STUDY** | **REFERENCES** | **COUNTRY/ POPULATION** | **OBJECTIVE** | **STUDY**  **DESIGN** | **RESULTS** |
| --- | --- | --- | --- | --- | --- |
| 1 | Al-Dubai SAR, Rampal KG. Prevalence and associated factors of burnout among doctors in Yemen. J Occup Health. 2010;52(1):58-65.  <http://doi.org/10.1539/joh.O8030> | Yemen  Physicians  (n = 563) | To determine the prevalence and factors associated with BS among Yemeni physicians. | Cross-sectional | EE was high in 63.2%, moderate in 17.2%, and low in 19.6% of the sample. About 19.4% had high DP, 24.6% had moderate, and 56.0% had low. About 33.0% had low PA, 25.4% had moderate, and 41.6% had high PA. A high degree of BS was identified in 11.7% of the studied population. |
| 2 | Alotni MA, Elgazzar SE. Investigation of Burnout, its associated factors and its effect on the quality of life of critical care nurses working in Buraydah Central Hospital at Qassim Region, Saudi Arabia. Open Nurs J.; 2020;14(1):190-202. <http://doi.org/10.2174/1874434602014010190> | Saudi Arabia  Nurses  (n = 170) | To investigate BS, associated factors, and impacts on quality of life among intensive care nurses working at the Central Hospital of Buraydah, Qassim region, Saudi Arabia. | Descriptive correlational study | EE was observed in 88.2% of the sample, and 82.4% presented high DP. PA was low in 94.1% of participants. Most nurses had a moderate level of BS according to the total score. |
| 3 | Silva, RADA, Teixeira AKM, Frota MMA, Maciel JAC, Farias MR. Job satisfaction and burnout among oral healthcare providers within the Unified Health System in Sobral, Ceará, Brazil. Rev Bras Med Trab. 2019;17(3):313-324. <http://doi.org/10.5327/Z1679443520190352> | Brazil  Dental surgeons, oral health technicians, and assistants  (n = 95) | To analyze job satisfaction and the prevalence of BS among oral health professionals in the public health care network of Sobral, Ceará, Brazil. | Cross-sectional | Participants presented a low overall prevalence (1.05%) of BS. In each subscale, low PA (37.9%), high EE (22.1%), and high DP (9.5%) were observed. The relation of professionals with at least one (53.6%) or two (6.3%) of the three subscales with high chances of BS was observed. |
| 4 | Amiri M, Khosravi A, Eghtesadi AR, et al. Burnout and its influencing factors among primary health care providers in the northeast of Iran. PLoS One. 2016. <http://doi.org/10.1371/journal.pone.0167648> | Iran  Primary healthcare providers  (n = 548) | To assess the level of BS among primary health care providers and identify its associated factors in northeastern Iran. | Cross-sectional | Of all participants, 35.7% had moderate to high levels of EE, 49.6% had high levels of lack of PA, and 8.8% had high levels of DP. BS mean score was 54.09 ± 27.23, and the mean scores of BS components were 15.48 ± 13.58 (EE), 3.71 ± 5.36 (DP), and 35.49 ± 13.54 (PA). About 41.8% of participants presented low BS levels, 52.7% suffered from moderate symptoms, and 5.5% suffered severe symptoms. |
| 5 | Beltrán CA, Baltazar RG, Moreno MP, Santacruz GH. Labor psychosocial risk factors, social support and Burnout syndrome in family doctors of three public health institutions, Guadalajara (México). Salud Uninorte. 2013;29(3):487-500. | Mexico  Physicians  (n = 410) | To analyze negative occupational psychosocial risks and social support as incidents of BS in family physicians working in three public health institutions in Guadalajara, Mexico. | Descriptive, analytical, and comparative design | Institution I showed  32.4% for EE and 16.2% for low PA. Institution II showed 11.9% for DP. The total prevalence was 43.5% for the institution I. |
| 6 | Beltrán CA, Salas JHB, Paredes JJR, Santacruz GH. Psychosocial variables and Burnout syndrome in general physicians in Tepic, Nayarit (México) Salud Uninorte. 2015;31(2):245-54. DOI:10.14482/sun.31.2.6018 | Mexico  Physicians  (n = 97) | To analyze the psychosocial factors associated with BS in general practitioners working in a public health institution in Tepic, Nayarit, Mexico. | Cross-sectional | The prevalence by domain was 9.3% for EE and 26.8% for lack of PA. No values were found for DP. The overall prevalence of BS was 32%. |
| 7 | Barroso SM, Guerra, ARP. Burnout and quality of life in health comunitary agents of Caetanópolis (MG) Cad. Saúde Colet. 2013;21(3):338-45. | Brazil  Community health agents  (n = 24) | To assess BS and quality of life of community health agents in Caetanópolis, Minas Gerais, Brazil. | Exploratory and correlational | All participants had BS. Most presented a moderate level of EE (58.3%), a high level of DP (54.2%), and a high level of lack of PA (58.3%). |
| 8 | Bijari B, Abassi A. Prevalence of burnout syndrome and associated factors among rural healthworkers (Behvarzes) in South Khorasan. Iran Red Crescent Med J. 2016;18(10). http://doi.org/10.5812/ircmj.25390 | Iran  Primary healthcare (rural) workers  (n = 423) | To determine the prevalence of BS and associated factors among rural health workers in health centers of Birjand University of Medical Sciences. | Cross-sectional | About 34.5% of participants presented moderate to severe levels of BS: 31.4% had EE, 16.8% had DP, and 47% had PA. Approximately 34.5% of participants had vocational BS, and 5.7% had severe vocational BS. |
| 9 | Bojčeski O, Galjak M, Kulić L, Đurić S, Mirković M, Milošević J. Testing the correlation between occupational stress and occupational burnout among nurses working in public health institutions. Praxis Med. 2019;48(3–4):13-18. http://doi.org/10.5937/pramed1904013b | Macedonia  Nurses  (n = 60) | To examine the relationships between occupational stress and occupational BS in nurses working in public healthcare institutions. | Not identified | Participants had a moderate level of EE (M=24.05), low DP (M=3.83), and low level of lack of PA (M=38.71). |
| 10 | Bressi C, Porcellana M, Gambini O, et al. Burnout among psychiatrists in Milan: a multicenter survey. Psychiatr Serv. 2009;60(7):7-10. http://doi.org/10.1176/appi.ps.60.7.985 | Italy  Physicians  (n = 81) | To determine the prevalence of BS and estimate the psychiatric morbidity and job satisfaction among psychiatrists in Milan. | Cross-sectional | Scores indicated high levels of EE in 49% of participants. High level of DP and low level of PA were observed in 39% and 22% of physicians, respectively. |
| 11 | Maciel JAC, Farias MR, Sampaio JJC, Guerrero JAP, Castro-Silva II. Professional satisfaction and prevalence of burnout syndrome in primary care oral health teams  in Sobral, Ceará-BrazilSalud Trab. 2018;26(1):34-44. | Brazil  Dental surgeons and oral health technicians/  assistants  (n = 50) | To analyze job satisfaction and determine the prevalence of BS in oral primary health care teams in Sobral, Ceara, Brazil. | Cross-sectional | For all professionals, the prevalence of the syndrome was low (2%, with only one case among oral health technicians). PA (high: 64%) was the subscale with the highest prevalence compared with EE (high: 26%) and DP (high: 16%). |
| 12 | Silva ATC, Lopes CS, Susser E, Coutinho LMS, Germani ACCG, Menezes PR. Burnout among primary health care workers in Brazil: results of a multilevel analysis. Int Arch Occup Environ Health. 2021;94(8):1863-75. http://doi.org/10.1007/s00420-021-01709-8 | Brazil  Physicians,  nurses, and community health agents  (n = 2940) | To determine the prevalence of BS in primary care teams, including community health workers. To identify the associations between BS and characteristics of individuals, team factors, and primary healthcare unit. | Cross-sectional | About 11.4% of participants presented severe BS. Physicians and community health workers presented the highest levels of BS. |
| 13 | Cruz SP, Abellán MV. Professional burnout, stress and job satisfaction of nursing staff at a university hospital. Rev Lat-Am Enferm. 2015;23(3):543-52. http://doi.org/10.1590/0104-1169.0284.2586 | Spain  Nurses and nursing assistants  (n = 258) | To describe the social and work characteristics of the nursing staff and assess BS and job satisfaction. | Cross-sectional | Moderate EE, high DP, and low PA were identified among participants. |
| 14 | Fuente-Solana EI, Pradas-Hernández L, González-Fernández CT, et al. Burnout syndrome in paediatric nurses: a multi-centre study. Int J Environ Res Public Health. 2021;18(3):1-11. http://doi.org/10.3390/ijerph18031324 | Spain  Nurses  (n = 95) | To determine the prevalence and levels of BS experienced by nurses. | Cross-sectional | About 38.6% of all participants presented high levels of BS. EE was low in 46.2%, moderate in 31.8%, and high in 22.0% of the sample.  For DP, 50% presented a low level, 31.5% were moderate, and 18.5% high.  For PA, a low level was found in 39.6%, moderate in 31.8%, and high in 28.6% of the sample. |
| 15 | Ferreira NN, Lucca SR. Burnout syndrome in nursing assistants of a public hospital in the state of São Paulo Rev Bras Epidemiol. 2015;18(1):68-79. http://doi.org/10.1590/19805497201500010006 | Brazil  Nursing technicians  (n = 538) | To determine the prevalence of BS in nursing technicians of a public university hospital and its association with sociodemographic and professional variables. | Cross-sectional | About 23.6% of participants presented high EE, 21.9% had high DP, and 29.9% had low PA. About 5.9% had all three domains suggestive of BS. |
| 16 | Engelbrecht MC, Bester CL, Van Den Berg H, Van Rensburg HCJ. A study of predictors and levels of burnout: the case of professional nurses in primary health care facilities in the free state. S Afr J Econ. 2008;76(Suppl.1):S5-S27. http://doi.org/10.1111/j.1813-6982.2008.00164.x | South Africa  Nurses  (n = 543) | To determine the prevalence of BS in public health professional nurses. To compare BS levels between nurses responsible for the antiretroviral treatment program and nurses working in primary health care units not providing antiretroviral treatment. To determine the influence of different work-related stressors on BS levels. | Not identified | About 68.7% and 85.1% reported high levels of EE and DP, respectively, while 91% reported moderate levels of PA. |
| 17 | Gan Y, Jiang H, Li L, Yang Y, Wang C, Liu J, et al. Prevalence of burnout and associated factors among general practitioners in Hubei, China: a cross-sectional study. BMC Public Health. 2019;19(1):1-9. http://doi.org/10.1186/s12889-019-7755-4 | China  Physicians  (n = 1015) | To determine the prevalence of BS and associated factors in general practitioners. | Cross-sectional | Of all participants, 24.83% reported high levels of EE, 6.21% reported high levels of DP, and 33.99% reported low PA. Overall, 35% scored high for BS in one dimension, 21% in at least two dimensions, and 2.46% scored high for all three dimensions. |
| 18 | González-Rodríguez R, López-Castedo A, Pastor-Seller E, Verde-Diego C. Burnout syndrome in the health system: the case of social health workers. Enferm Glob, 2020;19(2):152-61. http://doi.org/10.6018/eglobal.382631 | Spain  Social workers  (n = 58) | To determine the prevalence of BS in health social workers working in a public health service in Galicia, Spain. | Cross-sectional | Participants had a high prevalence of EE and DP, and a low prevalence of PA. |
| 19 | Grau A, Flichtentrei D, Suñer R, Prats M, Braga F. Influence of personal, professional and cross-national factors in Burnout Syndrome in Hispanic Americans and Spanish Health Workers (2007)Rev Esp Salud Publica. 2009;83(2):215-30. http://doi.org/10.1590/S1135-57272009000200006 | Spain; Argentina; Uruguay; Mexico; Ecuador;  Peru; Colombia; Guatemala;  El Salvador  Physicians,  nurses, psychologists, dentists, and nutritionists  (n = 11530) | To determine the prevalence of BS among healthcare professionals in Spanish-speaking countries and explore its association with sociodemographic, professional, and personal characteristics. | Not identified | The prevalence of BS was 14.9% in Spain, 14.4% in Argentina, and 7.9% in Uruguay. Participants from Mexico, Ecuador, Peru, Colombia, Guatemala, and El Salvador had prevalence rates between 2.5% and 5.9%. Physicians had a prevalence of 12.1% and nurses 7.2%; dentists, psychologists, and nutritionists presented prevalence below 6%. |
| 20 | Grau A, Suñer R, García MM. Burnout syndrome in health workers and relationship with personal and environmental factors. Gac Sanit. 2005;19(6):463-70. http://doi.org/10.1016/s0213-9111(05)71397-2 | Spain  Physicians,  nurses, and technicians  (n = 1095) | To determine the prevalence of occupational BS in hospital health care workers and assess its relationships with personal and environmental factors. | Cross-sectional | About 41.6% of participants had a high EE, mainly doctors and the nursing staff. Also, 23% had high levels of DP (mainly doctors), and 27.9%, had low PA (mainly technicians and doctors). |
| 21 | Hayter M. Burnout and AIDS care-related factors in HIV community clinical nurse specialists in the North of England. J Adv Nurs. 1999;29(4):984-93. http://doi.org/10.1046/j.1365-2648.1999.00973.x | England  Nurses  (n = 32) | To determine the prevalence and nature of BS among nurse specialists in HIV care. To identify the relationship between BS and HIV  specific factors. | Quantitative, Qualitative, Exploratory, and Descriptive | About 44% scored high to moderate on EE, and 27% scored high. For PA, 47% scored low to moderate, and 20% scored low. For DP, 97% scored low, and 3% scored moderate; scores classified as high were not observed. |
| 22 | Hui W, Li L, Yang W, Fei G, Xue Z,Wang L. Factors associated with burnout among Chinese hospital doctors: a cross-sectional study. BMC Public Health.2013;13(1):786.  http://doi.org/10.1186/1471-2458-13-786 | China  Physicians  (n = 1202) | To investigate the association of demographics, occupational stress, and work situation with BS. | Cross-sectional | BS mean scores were 11.46 (7.51%) for EE, 6.93 (5.15%) for DP, and 24.07 (9.50%) for PA. |
| 23 | Khan N, Palepu A, Dodek P, et al. Cross-sectional survey on physician burnout during the Covid-19 pandemic in Vancouver, Canada: the role of gender, ethnicity and sexual orientation. BMJ Open. 2021;11(5):e050380. http://doi.org/10.1136/bmjopen-2021-050380 | Canada  Physicians  (n = 302) | To determine the prevalence of BS in physicians during the COVID-19 pandemic and correlate with gender, ethnicity, or sexual orientation. | Cross-sectional | Burnout prevalence was 68% among all participants: 71% for females and 64% for males. BS was reported by 60% of LGBTQ participants. For ethnicity, the prevalence of BS was 71% for white physicians, 68% for South Asian physicians, 78% for physicians identified as other ethnicities, and 54% for Asian and Asian Pacific Islander physicians. |
| 24 | Koval KW, Lindquist B, Gennosa C, et al. First look at emergency medical technician wellness in India: application of the Maslach Burnout Inventory in an unstudied population. PLoS ONE. 2020;15(3):1-14. http://doi.org/10.1371/journal.pone.0229954 | India  Physicians  (n = 296) | To determine the prevalence of BS among emergency physicians in India. | Exploratory, Descriptive | Burnout prevalence was 28.7%. Participants presented high levels of PA and EE and moderate levels of DP. |
| 25 | Lahana E, Papadopoulou K, Roumeliotou O, Tsounis A, Sarafis P, Niakas D. Burnout among nurses working in social welfare centers for the disabled. BMC Nurs. 2017;16(1):1-10.  http://doi.org/10.1186/s12912-017-0209-3 | Greece  Nurses  (n = 180) | To investigate BS and associated factors among nurses working with people with intellectual disabilities. | Cross-sectional | Participants had high levels of EE (mean = 31.36) and DP (mean = 11.27), and low levels of PA (mean = 44.02). |
| 26 | Lasebikan VO, Oyetunde MO. Burnout among nurses in a nigerian general hospital: prevalence and associated factors. ISRN Nurs. 2012;1-6.  http://doi.org/10.5402/2012/402157 | Nigeria  Nurses  (n = 270) | To determine the prevalence and associated factors of BS among nurses in a Nigerian general hospital. | Not identified | A high level of BS was identified in 39.1% of participants for the EE domain, 29.2% for DP, and 40.0% for lack of PA. |
| 27 | Lee FJ, Stewart M, Brown JB. Stress, burnout, and strategies for reducing them What’s the situation among canadian family physicians? Can Fam Physician. 2008;54(2):234-35. | Canada  Physicians  (n = 158) | To assess the levels of stress and BS among Canadian family physicians and identify management strategies. | Census | About 47.9% of participants had high levels of EE, 46.3% had high DP, and 47.9% had low PA. |
| 28 | Lesić AR, Stefanovic NP, Perunicić I, Milenković P, Tosevski DL, Bumbasirević MZ. Burnout in Belgrade orthopaedic surgeons and general practitioners, a preliminary report. Acta Chir Iugosl. 2009;56(2):53-9. http://doi.org/10.2298/ACI0902053L | Serbia  Physicians  (n = 68) | To assess the level of BS in orthopedic surgeons and general practitioners and its associations with demographic and job characteristics. | Not identified | About 70% of participants had high EE. Orthopedic surgeons had a slightly higher level of DP (55%) than general practitioners (38%). General practitioners had lower PA (48%) than orthopedic surgeons (29%). |
| 29 | Li H, Zuo M, Gelb AW, Zhang B, Zhao X, Yao D, et al. Chinese anesthesiologists have high burnout and low job satisfaction: a cross-sectional survey. Anesth Analg. 2018;126(3):1004-12. http://doi.org/10.1213/ANE.0000000000002776 | China  Physicians  (n = 2873) | To determine the incidence of BS in anesthesiologists and anesthesia residents. To measure job satisfaction and identify factors associated with BS. | Cross-sectional | Results indicated a prevalence of 57% (95% CI: 55 – 59) for high EE, 49% (95% CI: 47 – 51) for high DP, and 57% (95% CI: 55 – 58) for low PA. |
| 30 | Li H, Yuan B, Meng Q, Kawachi I. Contextual factors associated with burnout among Chinese primary care providers: a multilevel analysis. Int J Environ Res Public Health. 2019;16(19). http://doi.org/10.3390/ijerph16193555 | China  Physicians,  nurses, and  public health agents  (n = 951) | To analyze workplace contextual factors associated with BS among primary care providers in the Shandong province, China. | Cross-sectional | The overall prevalence of high EE (≥ 27 points) was equivalent to 33.12% (n = 315), 8.83% (n = 84) had high DP (≥ 13 points), and 41.43% (n = 394) had low PA (≤ 31 points). About 32.91% (n = 313), 19.77% (n = 188), and 20.50% (n = 195) of participants experienced, respectively, moderate levels of EE, DP, and low PA. Results revealed that 33.12%, 8.83%, and 41.43% of participants were experiencing a high level of EE and DP, and low PA. |
| 31 | Lorenz VR, Benatti MCC, Sabino MO. Burnout and stress among nurses in a university tertiary hospital. Rev Lat-Am Enferm. 2010;18(6). https://doi.org/10.1590/S0104-11692010000600007 | Brazil  Nurses  (n = 149) | To investigate BS in a tertiary university hospital and identify stressors in the work environment. | Cross-sectional | A total of 22.4% (quartile) and 33.3% (tercile) of nurses had high EE, while 21.5% (quartile) and 26.4% (tercile) of participants had high DP. Last, 27.8% (quartile and tercile) of the sample had low PA.  About 7.30% (quartile) and 10.22% (tercile) of nurses had high levels of EE and DP and low PA. |
| 32 | Lu S, Zhang L, Klazinga N, Kringos D. More public health service providers are experiencing job burnout than clinical care providers in primary care facilities in China. Hum Resour Health. 2020;18:1-11. http://dx.doi.org/10.1186/s12960-020-00538-z | China  Physicians,  nurses, and pharmacists.  (n = 17816) | To determine the prevalence of BS and identify the associated factors among clinical and public health care providers in primary care facilities. | Cross-sectional | Half of participants suffered from BS, and 2.99% presented severe BS. The presence of BS and severe BS in the primary care service group (58.06% and 5.25%, respectively) was higher than in the clinical care group (47.55% and 2.26%, respectively). The prevalence of low PA (40.85%) was higher than that of EE (14.60%) and DP (13.20%) among general providers. The prevalence of all three domains among primary care service providers was higher than among clinical care providers. |
| 33 | MacKanga JR, Mouloungui EGM, Iba-Ba J, Pottier P, Kombila JBM, Boguikouma JB. Burnout level and associated factors in a sub-Saharan African medical setting: Prospective cross-sectional survey. BMC Med Educ. 2020;20(1):295. http://dx.doi.org/10.1186/s12909-020-02194-2 | Gabon  Physicians  (n = 104) | To determine the prevalence of BS and associated factors in physicians of a sub-Saharan African country. | Cross-sectional | The prevalence of severe BS was 1.9%. The prevalence of BS symptoms was 34.6%: EE ≥27, 17.3%; DP ≥10, 25.0%; and PA ≤33, 7.7% (95% CI: 3.4 - 14.6%). |
| 34 | Marcelino GT, Cerveira JM, Carvalho I, Costa, JA, Lopes M, Calado NE, Marques-Vidal P. Burnout levels among portuguese family doctors: a nationwide survey. BMJ Open. 2012;2(3):e001050. http://dx.doi.org/10.1136/bmjopen-2012-001050 | Portugal  Physicians  (n = 150) | To determine the prevalence of BS in family physicians working in the Portuguese National Health System. | Cross-sectional | Overall, 25.3% of participants scored high for EE, 16.2% scored high for DP, and 16.7% scored low for PA. About 2.0% of participants scored high for all three dimensions. |
| 35 | Marchalik D, Brems J, Rodriguez A, Lynch JH, Padmore J, Stamatakis L, et al. The impact of institutional factors on physician burnout: a national study of urology trainees. Urology. 2019;131:27-35. https://doi.org/10.1016/j.urology.2019.04.042 | United States of America  Physicians  (n = 211) | To determine the prevalence of BS in urology interns and examine the influence of personal, programmatic, and institutional factors on BS levels. | Cross-sectional | Of all participants, 144 (68.2%) met the criteria for BS. Of participants without BS, 102 (48.3%) had high EE, 120 (56.9%) had high DP, and 78 (37.0%) had high DP and EE. |
| 36 | Nishimura Y, Miyoshi T, Sato A, Hasegawa K, Hagiya H, Kosaki Y, et al. Burnout of healthcare workers amid the covid-19 pandemic: a follow-up study. Int J Environ Res Public Health. 2021;18(21):11581. https://doi.org/10.3390/ijerph182111581 | Japan  Physicians and  nurses  (n = 130) | To examine the relationship between the COVID-19 pandemic and the prevalence of BS in health care workers in Japan. | Cross-sectional | No statistically significant differences in EE or PA scores were observed between participants that cared for COVID-19 patients or patients under investigation compared with those who did not care for COVID-19 patients. Among participants engaged with COVID-19 care in the past two weeks, six (50.0%) experienced BS. BS was observed in two (9.5%) of those who did not engage with COVID-19 care in the past two weeks. |
| 37 | Ogdon Lebrón MA, Díaz-Reissner CV. Burnout Syndrome in dentists working at the XVIII Sanitary Region of the Ministry of Public Health and Social, Paraguay. Rev Salud Publica Parag. 2017;7(1):21-7. https://doi.org/10.18004/rspp.2017.junio.21-27 | Paraguay  Dentists  (n = 83) | To determine the frequency of BS in dentists providing services in the XVIII Sanitary Region of the Ministry of Public Health and Social Security, Asunción, Paraguay. | Cross-sectional | The MBI-HSS indicated low levels in all domains, mainly for PA. The values obtained were 7.08 ± 10.53 for EE, 12.23 ± 16.99 for DP, and 3.12 ± 19 for PA. Only one dentist was classified with BS. |
| 38 | Okwaraji FE, Aguwa EN. Burnout and psychological distress among nurses in a Nigerian tertiary health institution. Afr Health Sci. 2014;14(1):237-45. https://doi.org/10.4314/ahs.v14i1.37 | Nigeria  Nurses  (n = 210) | To determine the prevalence of BS and psychological distress among nurses working in a Nigerian tertiary health institution. | Cross-sectional | A high level of BS was identified in 42.9% of participants for EE, 47.6% for DP, and 53.8% for low PA. BS was more present in nurses under 35, female, unmarried, and holding a nursing degree than graduated nurses working as nurse officers. |
| 39 | Osman D, Abdlrheem S. Burnout and Job satisfaction among healthcare providers in Aswan University Hospital, Upper Egypt. J High Inst Public Health. 2019;49(1):64-72. https://doi.org/10.21608/jhiph.2019.29468 | Egypt  Physicians and  nurses  (n = 283) | To determine BS levels and job satisfaction among healthcare workers at Aswan University Hospital. | Cross-sectional | Most participants (51.2%) had a high level of EE, and low PA was observed in 39%. A high level of DP was observed in approximately one-third (32.86%) of participants. The high EE among physicians was approximately 60% *versus* 43.6% among nurses (p=0.001). The rate of low PA was lower among physicians (10.4%) than nurses (28.9%) (p< 0.001). Physicians had higher DP (38%) than nurses (27.5%) (p= 0.001). |
| 40 | Paiva CE, Martins BP, Paiva BSR. Doctor, are you healthy? A cross-sectional investigation of oncologist burnout, depression, and anxiety and an investigation of their associated factors. BMC Cancer. 2018;18(1):1044. https://doi.org/10.1186/s12885-018-4964-7 | Brazil  Physicians  (n = 227) | To determine the prevalence of BS, anxiety, and depression among oncologists and identify potential pre-established characteristics. | Cross-sectional | About 58.1% of participants had BS. Regarding MBI domains, 41.9%, 37.6%, and 50.9% had high EE, high DP, and low PA, respectively. |
| 41 | Pantenburg B, Luppa M, König HH, Riedel-Heller SG. Burnout among young physicians and its association with physicians’ wishes to leave: results of a survey in Saxony, Germany. J Occup Med Toxicol. 2016;11(1):1-10. https://doi.org/10.1186/s12995-016-0091-z | Germany  Physicians  (n = 1784) | To assess BS among physicians and the association between BS symptoms and the willingness to leave clinical practice or go abroad for clinical work. | Cross-sectional | Males scored significantly higher than females in the DP domain. The domains were also categorized into low, moderate, and high BS. About 11% of participants had high scores of BS in all domains, and 34% did not score high scores in any domain. A higher proportion of males working full time or working with inpatient care had high scores in all domains. |
| 42 | Rachiotis G, Kourousis C, Kamilaraki M, Symvoulakis EK, Dounias G, Hadjichristodoulou C. Medical supplies shortages and burnout among greek health care workers during economic crisis: a pilot study. Int J Med Sci. 2014;11(5):442-7. https://doi.org/10.7150/ijms.7933 | Greece  Physicians and  nurses  (n = 303) | To investigate shortages of medical supplies in two Greek hospitals of the National Health System and assess their impact on the BS of health workers. | Cross-sectional | The prevalence of EE  DP, and low PA were 44.5%, 43.2%, and 51.5%, respectively. |
| 43 | Rajan S, Engelbrecht A. A cross-sectional survey of burnout amongst doctors in a cohort of public sector emergency centres in Gauteng, South Africa. Afr J Emerg Med. 2018;8(3):95-9. https://doi.org/10.1016/j.afjem.2018.04.001 | South Africa  Physicians  (n = 93) | To determine the level of BS among physicians in a cohort of public sector emergency centers in Gauteng, South Africa. | Cross-sectional | BS domains indicated a mean EE score of 31.69 (standard deviation, SD=10.32), with 62 respondents (66.7%) in the high-risk group and 86 (92.5%) in the moderate- to high-risk group. The mean DP score was 13.39 (SD=6.21), with 50 participants (53.8%) in the high-risk group, and 75 (80.7%) in the moderate- to high-risk group. The mean PA score was 34.87 (SD=6.54), with 21 participants (22.6%) in the high-risk group and 65 (69.9%) in the moderate- to high-risk group. |
| 44 | Renzi C, Tabolli S, Ianni A, Di Pietro C, Puddu P. Burnout and job satisfaction comparing healthcare staff of a dermatological hospital and a general hospital. J Eur Acad Dermatol Venereol. 2005;19(2):153-7. <https://doi.org/10.1111/j.1468-3083.2005.01029.x> | Italy  Physicians and  nurses  (n = 344) | To assess BS and job satisfaction among dermatologists and nurses working with dermatologic patients and compare with physicians and nurses from other specialties. | Not identified | The prevalence of EE  was significantly low among nurses working in dermatology compared with nurses from other specialties. |
| 45 | Rionda IS, Cortés-García L, Jiménez MVM. The role of burnout in the association between work-related factors and perceived errors in clinical practice among Spanish residents. Int J Environ Res Public Health. 2021;18(9):4931. https://doi.org/10.3390/ijerph18094931 | Spain  Physicians,  nurses, pharmacists, and psychologists  (n = 237) | To determine the level of BS among residents of different specialties. To identify work-related factors influencing BS. To explore the associations between work-related factors, BS, and perceived errors in clinical practice. To analyze the mediating role of BS in the relationships between work-related factors and perceived medical errors. | Not identified | Almost half of residents reported a high level of BS (N = 116, M = 65.91, SD = 9.58). Mean scores for each domain were as follows: 25.63 for EE (moderate BS), 10.88 for DP (high BS), and 29.4 for PA (high BS). More specifically, 44.7% of residents had high scores for EE, 53.6% had high DP scores, and 74.7% had low PA. |
| 46 | Tomljenovic M, Kolaric B, Stajduhar D, Tesic V. Stress, depression and burnout among hospital physicians in Rijeka, Croatia. Psychiatr Danub. 2014;26(Suppl 3):450-8. | Croatia  Physicians  (n = 286) | To determine the prevalence of depression and BS and observe their association with work stressors. | Cross-sectional | The high level of EE was the most frequent among the three groups of participants (surgical group 40.4%, non-surgical group 46.9%, and diagnostic group 38.5%). The prevalence of high EE was 43.6%. The surgical group had similar results regarding low levels (40.4% to 41.3%). DP was low in all  groups of participants (surgical group 47.7%; non-surgical group 49.3%; and diagnostic group 69.2%). The total prevalence of DP was more frequent in the low range (50.5%). PA was present at highest levels in all three groups (surgical group  42.2%; non-surgical group 51.3%; and diagnostic group 65.4%). The overall prevalence of PA was more frequent in the high range (49.1%). |
| 47 | Trindade LL, Lauter L. Syndrome of Burnout among the workers of the Strategy of Health of the FamilyRev Esc Enferm USP. 2010;44(2):274-9. | Brazil  Physicians, nurses, nursing technicians, dentists, dental assistants, and community health agents.  (n = 86) | To identify BS and associated factors among professionals working in the Family Health Strategy in Santa Maria, Rio Grande do Sul. | Descriptive | Mean scores in the three domains of the MBI were 9.0 ± 3.05 for DP, 23.87 ± 7.19 for EE, and 13.84 ± 4.82 for PA. Participants affected by BS scored one standard deviation above the mean for EE and DP domains and one standard deviation below the mean for PA. The group comprised six professionals, three community health agents, two nursing technicians, and one physician - all females aged between 21 and 40 years-old and working in different health units. |
| 48 | Vasconcelos EM, Martino MMF. Predictors of burnout syndrome in intensive care nurses Rev Gaucha Enferm. 2018;38(4):e65354. https://doi.org/10.1590/1983-1447.2017.04.65354 | Brazil  Nurses  (n = 91) | To determine the prevalence of BS in intensive care unit nurses and its predictors. | Cross-sectional | Of all participants, 78 (85.7%) did not present BS, and 13 (14.3%) did. Regarding the domains, 47.2% had high EE, 34.1% had high DP, and 34.1% had low PA. |
| 49 | Zarei E, Ahmadi F, Sial MS, Hwang J, Thu PA, Usman SM. Prevalence of burnout among primary health care staff and its predictors: a study in Iran. Int J Environ Res Public Health. 2019;16(12):2249. https://doi.org/10.3390/ijerph16122249 | Iran  Physicians and nurses  (n = 524) | To determine the level of BS and predictive factors among primary healthcare workers in western Iran. | Cross-sectional | Of all participants, 90.5% had high DP, 55.3% had high EE, and 98.9% had low PA scores. In addition, 52.9% (277 people) suffered from high BS. |
| 50 | Zha N, Patlas MN, Neuheimer N, Duszak Jr R. Prevalence of burnout among canadian radiologists and radiology trainees. Can Assoc Radiol J. 2018;69(4):367-72. https://doi.org/10.1016/j.carj.2018.05.005 | Canada  Physicians  (n = 262) | To assess BS among radiologists and radiology trainees in Canada. | Not identified | Results indicated low levels of BS in the PA domain, but high levels in the EE and DP domains. For the EE domain, 15.3% scored low, 13.0% scored moderate, and 71.8% scored high. For the DP domain, 36.3% scored low, 15.7% scored moderate, and 48.1% scored high. |
| 51 | Zhou Y, Gu W, Tao R, Chen C. The impact of gender and working hours on pulmonary physician burnout. Ann Transl Med. 2020;8(18):1166. https://doi.org/10.21037/atm-20-5827 | China  Physicians  (n = 125) | To evaluate personal and professional characteristics associated with BS in pulmonologists. | Not identified | Median scores for EE, DP, and PA were 25, 5, and 38, respectively. Of all participants, 44.8% had high EE, 27.2% had high DP, and 28.8% had low PA. Also, 48.8% had at least one symptom of BS. Female physicians (56.6%, 50/91) showed more BS than male physicians (32.3%, 11/34) (P = 0.025). |
| 52 | Kosan Z, Calikoglu EO, Guraksin A. Levels of burnout and their associated factors among physicians working in Northeast Anatolia. Niger J Clin Pract. 2018;21(7):875-81. https://doi.org/10.4103/njcp.njcp_298_17 | Turkey  Physicians  (n = 711) | To determine BS levels and associated factors in physicians in Erzurum, Anatolia, Turkey. | Cross-sectional | Mean MBI scores were 15.6 ± 7.0 for EE, 5.7 ± 3.9 for DP, and 21.0 ± 4.4 for PA. Mean scores for EE and DP were significantly high, while those for PA were significantly low in physicians younger than 25 years, not taking a vacation, working in public hospitals, and working as research assistants. BS levels among participants were low (EE in 75% of participants, DP in 76.2%, and low PA in 69.6%). |
| 53 | Khalafallah AM, Lam S, Gami A, et al. Burnout and career satisfaction among attending neurosurgeons during the COVID-19 pandemic. Clin Neurol Neurosurg. 2020;198:106193. https://doi.org/10.1016/j.clineuro.2020.106193 | United States of America  Physicians  (n = 407) | To investigate the impacts of the pandemic on BS and career satisfaction among neurosurgeons. | Not identified | The overall rate of BS was  20.4%. Results indicated low levels of EE (51.6%), low levels of DP (87.5%), and high levels of PA (81.1%). |
| 54 | Cañadas-De la Fuente GA, Albendín-García L, Cañadas GR, San Luis-Costas C, Ortega-Campos E, De la Fuente-Solana EI. Nurse burnout in critical care units and emergency departments: intensity and associated factors. Emergencias. 2018;30(5):328-31. | Spain  Nurses  (n = 337) | To determine the level of BS among nurses in intensive care units and emergency departments. To analyze the relationships between BS and sociodemographic, labor, and psychological factors. | Not identified | High levels of BS were identified in 38.5% of participants. EE was present in 10.5% of participants, DP in 16.8%, and low PA in 63.3%. |
| 55 | Mota CM, Dosea GS, Nunes OS. Assessment of the prevalence of burnout syndrome in community health agents of the city of Aracaju in the state of Sergipe, Brazil. Ciên Saúde Colet. 2014;19(12):4719-26.  https://doi.org/10.1590/1413-812320141912.02512013 | Brazil  Community health agents  (n = 222) | To determine the presence of BS in community health agents in Aracaju, Sergipe, Brazil. | Cross-sectional | Of all participants, 57.7% had moderate or severe scores of EE, 51.8% had moderate or severe scores of DP, and 59% had moderate to high PA at work. About 59.9% of participants showed no tendency to develop BS, 10.8% showed a moderate tendency, and 29.3% showed characteristics compatible with BS. |

BS: Burnout syndrome; DP: depersonalization; EE: emotional exhaustion; PA: personal accomplishment; MBI: Maslach Burnout Inventory; MBI-HSS: Maslach Burnout Inventory Human Services Survey.
